# Education Research: Neurologic Education in Nurse Practitioner Programs: Results of a National Needs Assessment Survey

**DOI:** 10.1212/NE9.0000000000200190

**Published:** 2025-02-05

**Authors:** Kathryn Swider, Calli Cook, Justin DiLibero, Kathryn E. Hall, Margaret Naclerio, Galina Gheihman, Lucy Morse, Andrew Busler, Elizabeth O'B. Woods, Matthew Walsh, Christopher T. Doughty, Daniel Harrison

**Affiliations:** 1 Division of Neurology, Lahey Hospital and Medical Center, Burlington, MA;; 2 Emory Brain Health Center, Atlanta, GA;; 3 Zvart Onanian School of Nursing, Rhode Island College, Providence;; 4 MGH Institute of Health Professions, Boston, MA;; 5 Neurology Department, Brigham and Women's Hospital, Boston, MA;; 6 Department of Neurology, Mass General Brigham, Harvard Medical School, Boston, MA;; 7 Department of Neurology, Mass General Brigham, Boston, MA;; 8 Department of Neurology, Massachusetts General Hospital, Boston, MA;; 9 Brigham and Women's Hospital Department of Neurology, Harvard Medical School, Boston, MA; and; 10 Department of Neurology, Boston University Chobanian & Avedisian School of Medicine, Boston, MA.

## Abstract

**Background and Objectives:**

A growing number of nurse practitioners (NPs) are entering neurologic practice. Postgraduate educational needs of NPs are partly dictated by previous neurologic educational experience, which is not well defined. We aim to describe neurologic education in NP programs to better inform efforts to develop tailored educational initiatives.

**Methods:**

A website review was performed to identify accredited NP programs and program leaders. A previously developed survey designed to facilitate a description of neurologic education in physician assistant (PA) programs was adapted for NP program leaders by an iterative approach. The survey was distributed in spring and fall 2023.

**Results:**

Three hundred and thirty-three Family NP, 140 Adult-Gerontologic Primary Care NP, and 136 Adult-Gerontologic Acute Care NP registered programs were identified. Leaders of 206 programs completed the survey (response rate = 34.9%). Two hundred four respondents (99.0%) reported neuroscience didactics included within their curriculum, typically contained within core, discipline-based courses. Neurologic disease and examination (n = 201, 98%) were covered most while lesion localization (n = 37, 18.0%) and neuroradiology (n = 50, 24.3%) were taught least often. Of 201 respondents who indicated that neurologic examination is included, 175 (87.1%) reported a hands-on approach. Didactic neuroscience instructors were specialized in clinical neurology in 109 programs (53.4%). One hundred sixty-nine programs (82.0%) offer a neurology clinical rotation, typically as an elective. Of respondents who provided an estimate, most reported that 10% or fewer of their students complete a neurology clinical rotation per year (n = 62, 77.5%). The most frequently reported barrier to offering clinical placement in neurology was lack of neurology preceptors (n = 85, 56.6%). Programs reporting graduates pursuing careers in neurology were associated with larger size, acute care track, and neurology NP as a didactic neuroscience instructor.

**Discussion:**

Lesion localization and neuroradiology are important targets for postgraduate training of NPs entering neurologic practice. Recruiting neurology NPs to teach neuroscience didactics may increase programs with students pursuing careers in neurology. Availability of clinical neurology rotations is not universal in NP programs. Encouraging neurology clinicians, including PAs and physicians, to precept NP students could help increase access to clinical neurology rotations. These results highlight important opportunities for augmenting neurologic education during and after NP programs.

## Introduction

Advanced practice providers (APPs), including nurse practitioners (NPs), are essential contributors to the care of patients with neurologic disease.^1,2^ There are over 380,000 NPs licensed in the United States as of 2022,^3^ and this number is projected to grow by nearly 50% by the year 2033.^4^ APPs report that neurologic education within degree programs is limited and express a need for tailored postgraduate neurologic training as they enter practice.^5^ Efforts to promote postgraduate APP education are rapidly growing, including postgraduate residency/fellowship programs and formal on-the-job training.^6,7^ Success of these programs depends on an accurate understanding of neurology education in APP degree programs.

NP degree programs are nationally accredited by multiple organizations. These groups define the required standards of education that are expected of accredited NP programs but do not specify organ systems or didactic topics that curricula must include.^8,9^ When enrolling in a program, students select a specialty track, such as Family NP (FNP), Adult-Gerontologic Primary Care NP (AGPCNP), or Adult-Gerontologic Acute Care NP (AGACNP). Because of the different patient populations these specialties serve, NP specialty tracks vary in course content and clinical requirements. NP programs may in part develop their curriculum based on certifying board requirements, but again, requirements vary between tracks. There are multiple certifying organizations through which NP program graduates can become licensed. Examination handbooks help outline expected examination content, but only some of these resources list “neurology” among themes covered by the examination and generally do not otherwise delineate specific neurologic topics or specify what percentage of their examination is made up of neurology content.^10-14^

Because of these factors, curricula between NP programs and within program tracks are quite heterogeneous. To better inform the educational needs of NPs entering neurologic practice, we aimed to describe didactic and clinical neurologic education in NP programs. In addition, we aimed to identify factors of NP programs that predict graduates entering neurology practice.

## Methods

The National Organization for Nurse Practitioner Faculties (NONPF) includes accredited member programs within the United States.^15^ Websites of NONPF-member programs were reviewed to identify programs with FNP, AGPCNP, and AGACNP tracks that were actively accepting students in 2023 and program leaders. These 3 tracks were selected because they are most likely to have graduates pursuing neurology specialties and co-managing patients with neurologic comorbidities as they enter practice.

A survey previously developed to describe neurologic education in physician assistant (PA) programs was adapted for NP program leaders, incorporating input from NP and physician neurology educators.^16^ The survey was administered using REDCap and was distributed to NP program leaders using email addresses collected through the website review (eAppendix 1). Participants responded to questions about their program's didactic neuroscience education, clinical neurology education, neurology preceptors, size, and graduates. When available, track coordinators were contacted first because they most likely oversee the specialty track directly. If a track coordinator was not listed on the website, program directors were then contacted because they oversee multiple tracks for the entire program. In programs without identifiable track coordinators or program directors, another leader with expected oversight over the NP program (e.g., graduate nursing director or dean) was invited to participate. Initial distribution occurred in spring 2023, and individuals from programs who had not responded were contacted again in fall 2023. Participants were offered the option to enter their name in a drawing for a chance to win one of fifty $50 gift cards after survey completion.

Quantitative survey results were reported using simple descriptive statistics. The effects of didactic and clinical formats, didactic instructor and clinical preceptor training, program size, and program track on the likelihood that a program reported graduates in neurology are reported with univariate odds ratios (ORs). In addition, logistic regression was performed using a stepwise solution with *p* < 0.05 set as criteria for entrance into and *p* > 0.10 set as criteria for removal from the model to explain the variance in programs with neurology graduates. Univariate ORs and logistical regression were implemented in SPSS version 28.0.0.0. Free response questions were analyzed using qualitative thematic analysis by 1 coder (K.S.) using an inductive approach. After initial codes and themes were generated, the coder met with other study team members (D.S.H. and C.T.D.) who were familiar with the data to review and define the final themes by consensus.

### Standard Protocol Approvals, Registrations, and Consents

This study was deemed to be of minimal risk and received exemption from full review from the Mass General Brigham Institutional Review Board (Protocol #2021P000404).

### Data Availability

Anonymized data not published within this article will be made available by request from any qualified investigator.

## Results

A total of 607 individual tracks from accredited NP programs were identified and included in the website review. The distribution of tracks was as follows: 331 FNP (54.5%); 140 AGPCNP (23.1%); and 136 AGACNP (22.4%). Contact information for at least 1 leader from 591 discreet program tracks was publicly available online, collected through the website review, and used for survey dissemination.

We received a total of 206 completed surveys (response rate of 34.9%) from 102 FNP (49.5%), 48 AGPCNP (23.3%), 52 AGACNP (25.2%), and 4 dual program (1.9%) tracks. Examples of dual tracks included combined FNP and AGACNP tracks. Respondents were program directors (n = 129, 62.6%), track coordinators (n = 59, 28.6%), or other program leaders (n = 18, 8.7%). Examples of titles of “other” respondents included “dean” and “school director.”

### Didactic Neuroscience Education

**Figure 1:**
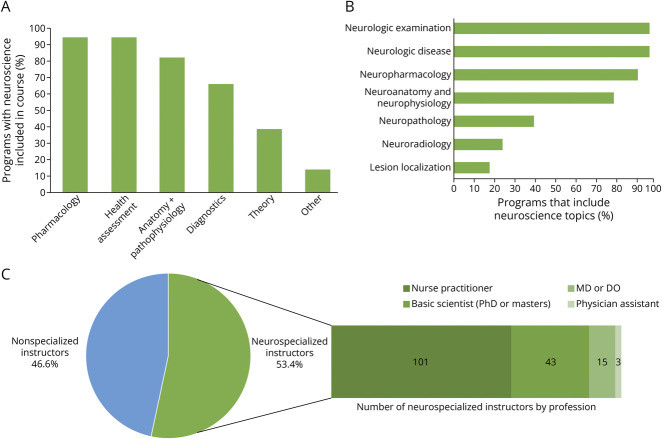
Characteristics of Didactic Neuroscience Education Within Nurse Practitioner Programs Courses in which neuroscience content is taught (A), topics included in curricula (B), and nonspecialized vs neurospecialized instructors in the pie chart form with breakdown of neurospecialized instructors based on profession in the bar chart form (C). Respondents were asked to select all that applied from a list of neurospecialized instructor types, accounting for a higher number of total neurospecialized instructors than the number of programs that reported they have neurospecialized instructors.

Three respondents reported a discrete neuroscience course (1.4%). One respondent (0.5%) reported that neuroscience is not included within the program curriculum. Most programs (n = 201, 97.6%) reported that neuroscience is taught within core classes of the curriculum. Neuroscience is most often taught in health assessment and promotion (n = 180, 94.2%), pharmacology (n = 181, 94.7%), and anatomy and pathophysiology (n = 157, 82.1%) courses (Figure 1A). Neuroscience is least often taught in diagnostics (n = 126, 65.9%) and theory (n = 74, 38.7%) courses.

**Figure 2:**
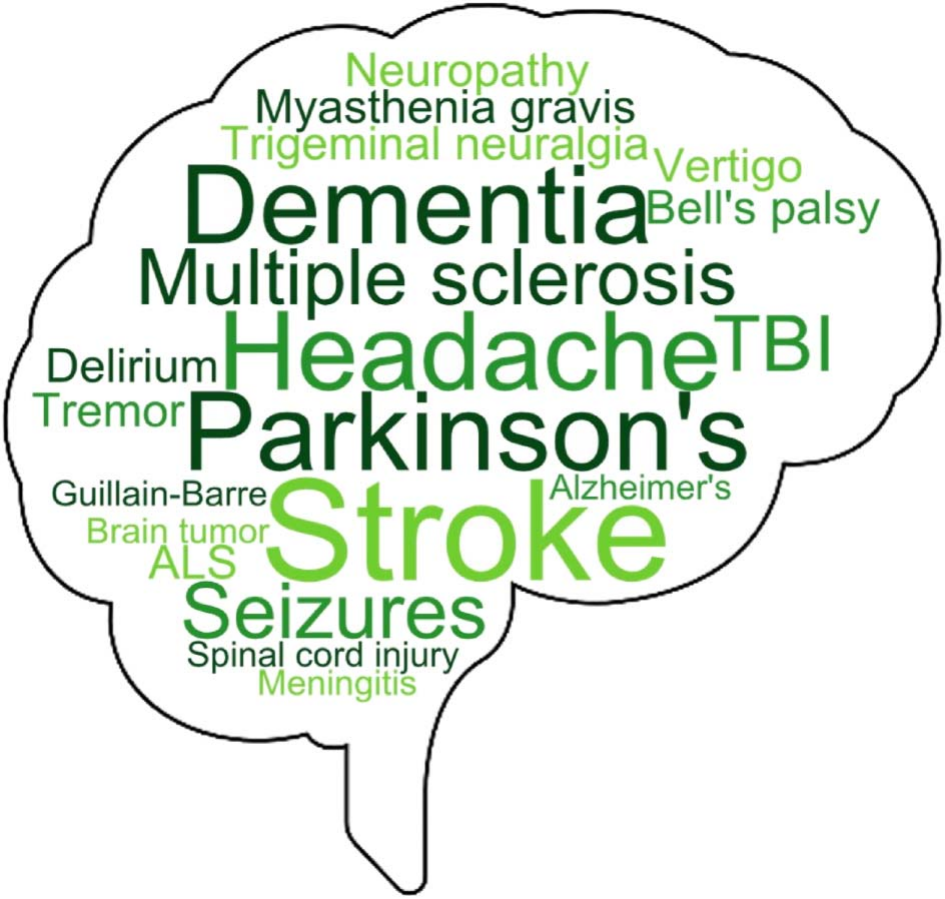
Common Neurologic Diseases Covered in Nurse Practitioner Program Curricula Larger words representing those covered relatively more frequently.

Neuroscience topics most often included within curricula were the neurologic examination (n = 201, 98%), neurologic disease (n = 201, 98%), neuropharmacology (n = 189, 92.1%), and neuroanatomy and neurophysiology (n = 163, 79.5%; Figure 1B). Neuroscience topics least often included were neuropathology (n = 92, 40%), neuroradiology (n = 50, 24.3%), and lesion localization (n = 37, 18%). Respondents who reported that neurologic disease is included in the curriculum were asked a follow-up, free response question to list the specific neurologic diseases covered. There were a total of 961 individual diseases listed, the most frequent of which were Parkinson disease (n = 64, 6.6%), headache (n = 58, 6%), and dementia (n = 55, 5.7%). Figure 2 presents a visual representation of neurologic diseases listed.

Two hundred four respondents (99.5%) provided information about their instructor profile for neuroscience didactics. Respondents from 109 programs (53.4%) reported neurospecialized faculty teaching neuroscience didactics (Figure 1C), including 101 programs (92.6%) with a neurospecialized NP, 43 programs (39.4%) with a neurologist, 15 programs (13.7%) with a basic scientist who is neurospecialized, and 3 programs (2.7%) with a neurospecialized PA (Figure 1C). These respondents were allowed to select multiple options from a list of neurospecialized instructor types, accounting for a higher number of total neurospecialized instructors (n = 162) than the number of programs that reported they have neurospecialized instructors (n = 109).

### Neurologic Examination Education

Of respondents who reported that neurologic examination teaching is included in their curricula, most (n = 175, 87.1%) indicated a hands-on approach. This included dedicated laboratory time for practice on other students (n = 153, 76.1%), use of standardized patients (n = 109, 54.2%), or other self-described hands-on methods (n = 5, 2.5%) such as “simulations” or “practice on medical colleagues.” To assess competence in performing the neurologic examination, respondents indicated using demonstration with a skills checklist (n = 165, 82%), a practical examination (n = 148, 73.6%), and/or preceptor assessment in the clinical setting (n = 136, 67.6%). Assessment with standardized patients (i.e., objective structured clinical examination) was less common (n = 114, 56.7%). Three respondents (1.4%) indicated that competence in performing a neurologic examination is not assessed.

### Clinical Neurology Education

**Figure 3:**
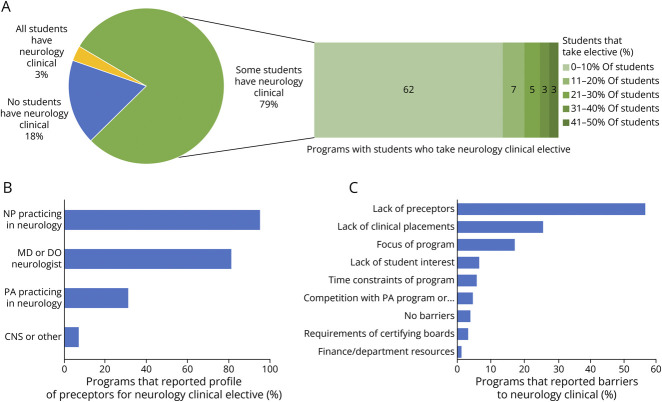
Characteristics of Clinical Neuroscience Education Within Nurse Practitioner Programs Structure of neurology clinical in the pie chart form with breakdown of percentage of students who take neurology clinical elective in the bar chart form (A), profile of neurology clinical preceptors (B), and reported barriers to offering neurology clinical (C).

All respondents were asked to describe how neurology clinical exposure is offered within their program (Figure 3A). Thirty-seven respondents (18%) reported that there is no opportunity for clinical exposure in neurology. Very few reported a required neurology clinical rotation for all students (n = 5, 3%). Most respondents (n = 169, 82.0%) indicated that some but not all students have a neurology clinical (by either elective or assignment). Of the programs in which a neurology clinical is offered as an elective, 62 respondents (77.5%) reported that 10% or fewer of their students elect to take the neurology clinical (Figure 3A). Of all programs that reported some form of clinical exposure in neurology, clinical preceptors included NPs who practice primarily in neurology (n = 161, 95.2%), neurologists (n = 138, 81.6%), PAs who practice primarily in neurology (n = 51, 31.3%), and clinical nurse specialists or other providers (n = 11, 7%; Figure 3B). Few respondents (n = 63, 37.5%) reported that their neuroscience didactic instructors also serve as preceptors. Of 150 respondents who reported barriers to offering neurology clinicals, the most often cited were lack of preceptors (n = 85, 56.6%) and lack of clinical placement sites (n = 39, 26%; Figure 3C).

### Characteristics of Graduates Pursuing Neurology Careers

Unadjusted odds of a program reporting graduates pursuing careers in neurology were higher in larger programs (OR 1.076, 95% CI 1.012–1.143, *p* = 0.02) and programs in which neurology NPs taught didactic neuroscience courses (OR 5.449, 95% CI 1.644–18.061, *p* = 0.0006; Table). Unadjusted odds of a program reporting graduates pursuing careers in neurology were lower in dual degree programs (OR 0.083, 95% CI 1.644–18.061, *p* = 0.048). The logistic regression model included neurology NPs as didactic instructors (adjusted OR [aOR] 4.272, 95% CI 1.194–15.279, *p* = 0.026), total number of graduates (aOR 1.090, 95% CI 1.020–1.166, *p* = 0.012), and AGACNP track (aOR 9.207, 95% CI 1.055–80.380, *p* = 0.045). The model was statistically significant (χ^2^ = 22.983, *p* < 0.001), explained 33.1% (Nagelkerke *R*^2^) of the variance in programs reporting neurology graduates, and correctly classified 86.0% of programs.

**Table:** Unadjusted Odds of a Program Reporting Graduates Pursuing Careers in Neurology by Variable

Variable	Odds ratio	95% CI	*p* Value
Total graduates	1.076	1.012–1.143	0.02
Track			
Acute care	7.484	0.946–59.181	0.056
Primary care	0.484	0.159–1.477	0.202
Family	0.964	0.342–2.722	0.945
Dual	0.083	0.007–0.977	0.048
Didactic instructor			
Neurology NP	5.449	1.644–18.061	0.006
Non-neurology NP	0.692	0.207–2.312	0.55
Neurology physician	1.231	0.367–4.135	0.737
Non-neurology physician	0.178	0.011–2.990	0.23
Basic scientist	2.2	0.265–18.261	0.465
Clinical format			
Elective	1.5	0.098–23.069	0.771
Required for some	3.1	0.257–37.451	0.373
Required for all	2.5	0.100–62.605	0.577
None offered	3.25	0.193–54.777	0.413
Preceptor training			
NP	1.411	0.352–5.647	0.627
PA	0.452	0.147–1.386	0.165
Physician	0.567	0.171–1.884	0.355

Abbreviations: NP = nurse practitioner; PA = physician assistant.

## Discussion

We report a review of neurologic education in NP programs. We found that while most NP programs incorporate neurology didactics into core classes in their curricula, some topics that are foundational for NPs entering neurology are less often included. In addition, neurology clinicals are not universally offered among programs and, when available, are typically offered as an elective. Many programs report that clinical availability is limited by lack of preceptors. Finally, graduates of NP programs with a neurology-specialized NP teaching didactic are more likely to enter the specialty. These results highlight neurology topics that postgraduate NP training should include, encourage opportunities to increase NP student access to neurology clinical rotations, and provide strategies for recruitment of NP students into neurologic practice.

As expected, NP curricula rarely include a dedicated neuroscience course because neurology didactics are more often included within core courses. This is because NP program curricula are universally discipline-based in contrast to a combination of organ system–based and discipline-based curricula present in medical schools and PA programs. The fundamental courses that comprise discipline-based NP curriculum are commonly referred to as the “3 Ps”: Pathophysiology, health assessment and Promotion, and Pharmacology.^17^ The 3 P courses often include topics such as neurologic disease, neurologic examination, neuropharmacology, neuroanatomy, and neurophysiology, which provide a foundational understanding of neurology. However, some topics that are essential to neurologic practice, such as lesion localization and neuropathology, are less often included within NP curricula. Neuroradiology in particular has been highlighted as a challenge for APPs entering neurology yet is infrequently included in the NP curriculum.^5,16^ NPs practicing in neurology are expected to recognize and localize symptoms, interpret the clinical relevance of neuroimaging findings, and diagnose patients with neurologic disorders. Postgraduate training should, therefore, focus on these topics, perhaps starting with localization, which is foundational to neurology practice. Proficiency in these areas is essential to ensuring NPs are successful clinicians in neurology.

Instruction on the neurologic examination was reported in nearly all programs. However, survey data from APPs entering neurology practice revealed that many APPs request additional teaching of the neurologic examination.^5^ This discrepancy may be related to the relative lack of clinical neurology exposure after didactic examination teaching. Most neurology clinicals are offered on an elective basis, which are not universally available among NP programs. The most frequently cited barrier to offering a neurology clinical was lack of preceptors. This is complicated by the fact that many NP programs require students to find their own preceptors. Encouraging neurology clinicians, including PAs and physicians, to precept NP students can increase access to clinical neurology rotations and provide students further opportunities to improve their neurologic examination skills. Precepting NP students may in turn be a valuable experience for neurologists who seek teaching opportunities.

A secondary aim of this study was to identify factors of NP programs associated with higher rates of graduates entering neurology practice. Our findings suggest that programs with neuroscience didactics taught specifically by neurology-trained NPs may increase the likelihood of graduates entering neurology. Instructors with neurology specialization, especially NPs, may serve as mentors and thus foster student interest in pursuing neurology careers after graduation. Didactic instructors who are specialized and have the expertise to connect neuroscience with clinical topics can further help to promote student interest in neurology. This has been shown to be an important strategy in combating “neurophobia” in medical students, although future work should aim to identify whether neurophobia is also present in NP students.^18^ Regardless, neurology NPs should be encouraged to become neuroscience instructors and support NP graduates entering the field of neurology.

One limitation of this study was the survey response rate. Although over 200 NP program leaders participated, this represents only 34% of all programs that were identified. It is possible that the relatively low response rate limits the generalizability of these findings to all NP programs. However, this was higher than a previous review of neurologic education in APP programs and augmented by survey incentives.^16^ Furthermore, the distribution of program tracks in the website review was similar to the distribution of program tracks represented by survey respondents, suggesting that the sample represents the underlying population. In addition, the survey was not truly anonymous because there was only 1 response per program and thus vulnerable to response bias. Finally, we did not collect information about what areas of neurology practice NP program graduates tend to pursue. Future studies should aim to determine the breakdown of NPs practicing neurology by subspecialty and practice setting, which could further inform development of educational resources.

Encouraging NPs to teach didactic neuroscience courses and all neurology clinicians to precept NP students are potential strategies for offering exposure to and fostering NP student interest in neurology. Neuroradiology and lesion localization are key targets for postgraduate training of neurology NPs. Supporting education and training for NP students and neurology NPs is critical for empowering this group of clinicians to provide excellent care for patients with neurologic disease.

## Glossary

AGACNP: Adult-Gerontologic Acute Care NP
AGPCNP: Adult-Gerontologic Primary Care NP
APP: advanced practice provider
aOR: adjusted OR
FNP: Family NP
NONPF: National Organization for Nurse Practitioner Faculties
NP: nurse practitioner
OR: odds ratio
PA: physician assistant
